# Persistent SARS-COV-2 RNA positivity in a patient for 92 days after disease onset: A case report

**DOI:** 10.1097/MD.0000000000021865

**Published:** 2020-08-21

**Authors:** Junxue Wang, Xiaofeng Hang, Bo Wei, Dingchen Li, Fangyan Chen, Wei Liu, Chaojie Yang, Xiaohui Miao, Li Han

**Affiliations:** a Department of Infectious Diseases, Changzheng Hospital, Second Military Medical University, Shanghai; b China PLA Institute for Disease Control and Prevention, Beijing, China.

**Keywords:** antiviral therapy, COVID-19, quarantine, SARS-CoV-2 nucleic acids

## Abstract

**Rationale:**

Recently, patients with COVID-19 who showed persistently positive SARS-CoV-2 nucleic acid test results despite resolved clinical symptoms have attracted a lot of attention. We report the case of a patient with mild symptoms of coronavirus disease (COVID-19), who achieved clinical recovery but showed persistently positive SARS-CoV-2 nucleic acid test results until Day 92 after disease onset.

**Patient concerns:**

The patient is a 50-year-old man with mild symptoms of coronavirus disease (COVID-19).

**Diagnoses:**

COVID-19 pneumonia.

**Interventions:**

The patient was quarantined for 105 days. Of these, inpatient quarantine lasted for 75 days. When the nucleic acid test results were negative for 3 consecutive days, the patient was discharged at Day 75 after disease onset. During this period, multiple samples were collected from the patient's body surface, the surrounding environment, and physical surfaces, but none of these tested positive for SARS-CoV-2. These samples included those from anal swabs, hands, inner surface of mask, cell phone, bed rails, floor around the bed, and toilet bowl surface. However, nucleic acid retest results on Day 80 and Day 92 after disease onset were positive for SARS-CoV-2 nucleic acids.

**Outcomes:**

The patient continued with quarantine and observation at home. After the test results on Days 101 and 105 after disease onset were negative, quarantine was terminated at last.

**Lessons:**

Per our knowledge, this is the longest known time that a patient has tested positive for SARS-CoV-2 nucleic acids. No symptoms were observed during follow-up. During hospitalization, the SARS-CoV-2 nucleic acid positivity was not observed in samples from the body surface and surrounding environment, and no verified transmission event occurred during the quarantine at home. After undergoing clinical recovery a minority of patients with COVID-19 have shown long-term positive results for the presence of the SARS-CoV-2 nucleic acid. This has provided new understanding and research directions for coronavirus infection. Long-term follow-up and quarantine measures have been employed for such patients. Further studies are required to analyze potential infectivity in such patients and determine whether more effective antiviral drugs or regimens to enable these patients to completely clear viral infection should be researched.

## 1 Introduction

The rapid spread of the SARS-CoV-2 (severe acute respiratory syndrome coronavirus 2, a novel coronavirus first observed at the end of 2019) has caused the World Health Organization to define coronavirus disease (COVID-19) as a public health emergency of international concern. In the span of several months, the number of patients with COVID-19 in the world has reached 4.8 million, and has continued to increase. Recently, the appearance of asymptomatic patients with COVID-19 has attracted attention.^[1–3]^ There are 3 possible scenarios for patients who are asymptomatic but test positive for viral nucleic acid: The first scenario is that the patient is infected, but without disease onset. In these patients, the SARS-CoV-2 nucleic acid test result is positive, showing that the patient is either in the incubation period, or has latent infection, or is a carrier. The second scenario is that the patient does not develop the disease, but laboratory tests show positivity for the SARS-CoV-2 antibody and negativity for the nucleic acid, suggesting the presence of subclinical infection. The third scenario is that the COVID-19-related symptoms have disappeared and the patient has entered the convalescent phase, but SARS-CoV-2 nucleic acid test results are positive for a long period of time.^[4]^ In this study, we report the case of a patient with COVID-19 who achieved clinical recovery but showed persistently positive results on the SARS-CoV-2 nucleic acid test for up to 92 days after disease onset. We studied the combined long-term treatment and management practice of this patient to analyze the clinical characteristics, pathogenesis, response to antiviral treatment, and infectivity in this case.

## 2 Case presentation

The patient described herein provided written informed consent to publish his case. A 50-year-old man who had symptoms of fever and dry cough for more than 1 month presented to the Huoshenshan Hospital on March 11, 2020. The patient developed low-grade fever without any obvious cause on January 22, 2020, with a peak temperature of 38.8°C, accompanied by mild cough; however, the patient was not tested for SARS-CoV-2 nucleic acids and was quarantined at home. During this period, he was treated with umifenovir, moxifloxcin hydrochloride, and Lianhua Qingwen capsules. His elevated body temperature returned to normal. On February 7, 2020, his wife developed COVID-19 and tested positive for SARS-CoV-2 nucleic acids. After treatment and recovery, she tested negative for these nucleic acids. On February 15, 2020, the patient tested positive for SARS-CoV-2 nucleic acids and he was admitted to the cabin hospital for treatment. Up until March 11, 2020, the patient still had mild cough and was admitted to the Huoshenshan Hospital for further diagnosis and treatment. After the onset of disease, his mental state, sleep, and diet remained fair, and bowel movements and micturition were normal. On admission, his temperature, pulse, respiration, and blood pressure were normal, and oxygen saturation (SpO_2_) in the absence of oxygen inhalation was 96%. Cardiopulmonary examination results were normal. After admission, the patient was diagnosed with COVID-19 (nucleic acid positive, moderate disease). Traditional Chinese medicine was used for antitussive and expectorant treatment, and his dry cough gradually disappeared. Computed tomography (CT) scans on March 11, 2020 showed the following imaging changes: Symmetrical bilateral chests, increased diffuse density shadows in both lungs, and fiber-like cords were seen. On March 24, 2020, his chest CT scans showed significant improved bilateral lung lesion, bullae formation in the right lower lung, and no other apparent abnormalities. Vital signs and SpO_2_ in the absence of oxygen inhalation (99%) were normal, and no abnormalities were found on physical examination.

However, the patient's nasopharyngeal swab test results were persistently positive for SARS-CoV-2 nucleic acids. During hospitalization, he was treated with drugs recommended per the Chinese guidelines. Oral umifenovir, recombinant human interferon nebulization, chloroquine phosphate, and intermittent convalescent plasma (1000 mL in total) were used successively. After the patient consented to participation in “the danoprevir & ritonavir clinical trial,” oral danoprevir and ritonavir were administered for 10 days. Subsequently, the patient tested negative for SARS-CoV-2 nucleic acids in his nasopharyngeal swabs for 3 consecutive days, and he was discharged at Day 75 after disease onset. We recommended that the patient continue with quarantine and observation at home, as well as wearing masks and maintaining ventilation. The patient was advised to maintain social distancing, eat alone, and practice good hand hygiene. However, viral nucleic acid tests on Day 5 after discharge (or Day 82 after disease onset) and Day 15 (or Day 92 after disease onset) showed positive results (shown in Table 1). After the test results on Days 101 and 105 after disease onset were negative, quarantine was terminated. During hospitalization, we obtained samples from other bodily sites, and the surrounding environment and objects, to test for viral nucleic acids. The results are shown in Table 2. Sputum samples were collected 4 times, and 3 of these tested positive. Stool samples were collected twice, and 1 tested positive. Anal swabs, and samples from the oral and nasal cavities, were obtained 4 times, and all tested negative. Additionally, all environmental samples tested negative. While the stool and sputum tested positive, SARS-CoV-2 nucleic acids were not detected in the nasal and oral cavities, hands, and inner mask surface. The surrounding environment, including the table at the head of his bed, pillows, bedside ground surfaces, cell phone surface (unwiped), and air filtration net in the room, tested negative for SARS-CoV-2 nucleic acids.

**Table 1:**
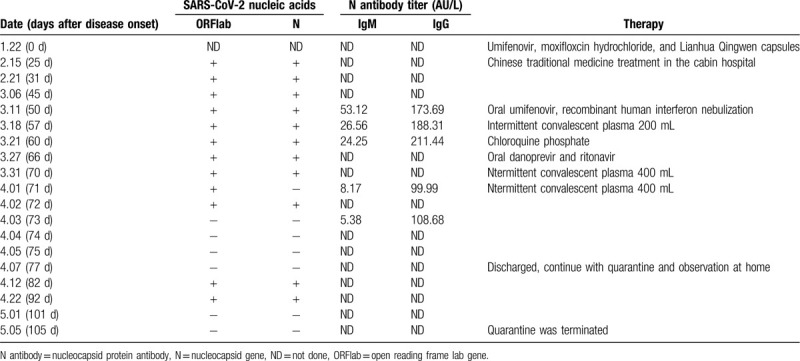
Results of SARS-CoV-2 nucleic acids and N antibody titer testing during therapy.

**Table 2:**
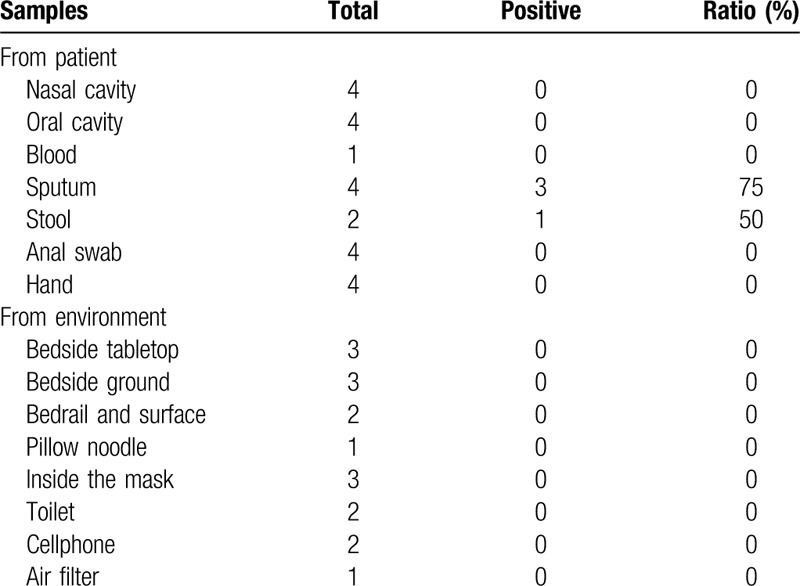
Results of SARS-CoV-2 nucleic acids testing of clinical and environmental samples.

## 3 Discussion

Recently, patients with COVID-19 who showed persistently positive SARS-CoV-2 nucleic acid test results despite resolved clinical symptoms have attracted a lot of attention. Li et al reported 1 patient who had persistently positive SARS-CoV-2 nucleic acid test results until Day 49 after disease onset.^[4]^ In our study, we have reported a patient who achieved clinical recovery but showed persistently positive SARS-CoV-2 nucleic acid test results until Day 92 after disease onset, which is currently the longest reported period for positive SARS-CoV-2 nucleic acid test results. The pathogenesis of this phenomenon is unclear, and the implications of these patients being potential sources of infection require further confirmation. It may also be pertinent to further examine the subsequent management and treatment of such patients.

A study using ultra-deep sequencing of 11 patient-derived virus isolates showed that there are 33 mutations in SARS-CoV-2, of which 19 are novel mutations.^[5]^ Among these patients, patient number 11 showed persistent positivity for viral nucleic acids for 45 days, and 3 nucleotide mutations were found in that particular virus isolate. Subsequent experiments analyzed the replication speed and pathogenicity, and suggested that persistent nucleic acid positivity may be associated with virus mutations. Another study reported a patient with COVID-19 who died due to stroke, and the nasopharyngeal swab samples from that patient tested negative for SARS-CoV-2 in 3 continuous polymerase chain reaction tests. However, electron microscopic and immunohistochemical staining (IHC) images showed the presence of residual SARS-CoV-2 in the patient's lungs. That study provided a new understanding of SARS-CoV-2 infection.^[6]^ The patient in our study tested negative for viral nucleic acids thrice in a row but tested positive during follow-up. This shows that even 2 consecutively negative results on the nucleic acid test cannot be used as a marker of in vivo viral clearance. This new data shows the possibility that infection may be persistent after clinical recovery in COVID-19 and even evolve to chronic infection. Peripheral blood lymphocytopenia is common in patients with COVID-19, and their autopsies show lymph nodal and spleen damage, suggesting that immune system damage may be a mechanism of persistent infection. There was no significant correlation between the in vivo SARS-CoV-2 N protein antibody changes and the nucleic acid test results seen in that patient. This may be because the N antibodies are not neutralizing antibodies. Regrettably, S antibody was not monitored at that time due to limitations. In addition, the interplay between the virus and immune system may lead to immune evasion or induced immune tolerance, which may need to be analyzed by further in-depth studies. These patients do not require long-term inpatient quarantine for their postdischarge management, as their infectivity is lower. Hence, it is recommended that they should quarantine at home for an extended period and undergo periodic SARS-CoV-2 nucleic acid tests, and should be observed for adverse outcomes due to persistent viral infection.

There is still a lot of debate on whether these patients are infectious and their infectivity. Positive viral nucleic acid test results show remaining viral genetic activity in these patients, and there have been reports of infectious viruses isolated from patient samples^[6]^; however, there has been no prior report suggesting that convalescent patients who test persistently positive for viral nucleic acids may infect others or cause local outbreaks. A recent study of 262 recovered patients found that there were 38 patients who tested positive for nucleic acids after recovery, but 21 close contacts of these patients tested negative, suggesting that they were not infected.^[7]^ A possible explanation is that such patients are often quarantined in hospitals, and individual and social preventive measures are adopted, which causes disease transmission to be lower. Conversely, in the case of our patient, although viral nucleic acid test results for sputum samples were positive on March 17 and 24, but his nasal and oral cavities, hands, and inner surface of mask all tested negative. His personal belongings and objects in his surrounding environment, including his cell phone, bed rail, floors, and toilet bowl surfaces, too tested negative, showing that the surrounding environment was not contaminated with the virus. This may be because this patient did not cough, expectorate, or show other behavior that could expel the virus from the respiratory tract, suggesting low possibility of infectivity.

As the prognoses and infectivity of patients with such persistent infection are unknown, it remains debatable whether an active search for effective antiviral treatment for such patients should be conducted. Currently, there are no drugs against the SARS-Cov-2 that have proven effective in clinical trials. Although remdesivir is considered the most promising antiviral drug, the local and overseas clinical trials have seen inconsistent results.^[8,9]^ This may be due to differences in selected timing for antiviral treatment, number of patients, and treatment endpoints. Our patient successively received different antiviral treatments as recommended in the Chinese guidelines, such as interferon nebulization, umifenovir, chloroquine phosphate, and convalescent phase plasma, but did not show persistent and stable negative nucleic acid test results. After he was hospitalized, he was treated with danoprevir and its booster, ritonavir, for 10 days. Subsequently, the patient tested negative for nucleic acid for 3 consecutive days. However, his nucleic acid test result was positive on Day 92 after discharge, showing that the aforementioned antiviral regimen did not have significant effects on the negative conversion of nucleic acid tests results. Thus, we should be vigilant for patients who may have chronic SARS-CoV-2 infection, and these patients should be closely followed up for a long period of time. We had examined the virus nucleic acid in the patient's nasopharyngeal swab samples frequently during hospitalization, and also in the surrounding environment, including the pillows, bedside ground surfaces, cell phone surface, and air filtration net in the room. However, the positive of virus nucleic acid does not mean the existence of infectious virus. Further studies such as identifying the infectivity of patient samples in infection cell and animal models should be done to elucidate the significance of these patients as source of infection. Although it was not shown if the patient shed infectious virus the entire time, this cannot be excluded and has to be taken into account in hygiene concepts.

## 4 Conclusions

In conclusion, after patients with COVID-19 have achieved clinical recovery, a minority of patients may show positive SARS-CoV-2 nucleic acid tests results for a long period of time. This provides new directions for research on SARS-CoV-2 infection. Further studies are required to understand the pathogenesis of this phenomenon and its implications in disease transmission. Such patients require long-term follow-up to observe potential long-term adverse outcomes, and they should be quarantined. Additionally, further studies are also required to analyze potential infectivity in such cases and determine whether a search for more effective antiviral drugs or regimens that completely clear the virus is warranted.

## Acknowledgment

The authors are grateful to the patient, who gave his informed consent for publication.

## Author contributions

**Conceptualization:** Xiaofeng Hang, Li Han, Junxue Wang.

**Data curation:** Bo Wei, Dingchen Li, Xiaofeng Hang.

**Investigation:** Junxue Wang, Wei Liu, Fangyan Chen.

**Supervision:** Xiaohui Miao, Li Han.

**Validation:** Xiaofeng Hang, Chaojie Yang

**Writing – original draft:** JunXue Wang, Xiaofeng Hang.

**Writing – review & editing:** JunXue Wang, XiaoFeng Hang.
